# Clinical predictive factors of pathologic complete response in locally advanced rectal cancer

**DOI:** 10.18632/oncotarget.8133

**Published:** 2016-03-16

**Authors:** Francesca De Felice, Luciano Izzo, Daniela Musio, Anna Lisa Magnante, Nadia Bulzonetti, Federico Pugliese, Paolo Izzo, Pierfrancesco Di Cello, Pietro Lucchetti, Sara Izzo, Vincenzo Tombolini

**Affiliations:** ^1^ Department of Radiotherapy, Policlinico Umberto I “Sapienza” University of Rome, Rome, Italy; ^2^ Department of Surgery “Pietro Valdoni”, Policlinico Umberto I “Sapienza” University of Rome, Rome, Italy; ^3^ Spencer-Lorillard Foundation, Rome, Italy

**Keywords:** rectal cancer, concomitant treatment, radiotherapy, neoadjuvant, athologic complete response

## Abstract

**Background:**

Predictive factors of pathologic complete response (pCR) after neoadjuvant chemoradiotherapy (nCRT) in locally advanced rectal cancer (LARC) are still not identified. The purpose of this study was to define them.

**Materials and Methods:**

Data from consecutive LARC patients treated between January 2008 and June 2014 at our Institution were included in the analysis. All patients were treated with a long course of nCRT. Demographics, initial diagnosis and tumor extension details, as well as treatment modalities characteristics were included in the univariate and logistic regression analysis.

**Results:**

In total 99 patients received nCRT, of whom 23 patients (23.2%) achieved pCR. Patients with and without pCR were similar in term of age, sex, comobidities, BMI and tumor characteristics. Multivariate logistic regression indicated that pre-treatment tumor size ≤ 5 cm was a significant predictor for pCR (p = 0.035), whereas clinical N stage only showed a positive trend (p = 0.084).

**Conclusions:**

Tumor size at diagnosis could be used to predict pCR, and thus to individualize therapy in LARC patients management. Validation in other studies is needed.

## INTRODUCTION

Neoadjuvant chemoradiotherapy (nCRT) is the standard treatment in locally advanced rectal cancer (LARC), due to the proven benefit in term of local recurrence and sphincter preservation [1–2]. Based on the observation that a greater tumor regression to nCRT resulted in superior survival, nowadays pathologic complete response (pCR) rate is becoming a focus of interest [3]. A recent meta-analysis has suggested that patients with pCR after nCRT had significantly better long-term clinical outcomes than did those with no-pCR, with 5-years rate of overall survival, disease free survival and risk for local recurrence of 87.6%, 83.3% and 2.8%, respectively [4]. It is important to note that there is no consensus of independent predictive factors for achieving pCR. Evidence of predictive value should be considered in order to optimize and individualize treatment approach. Nowadays it is assumed that all LARC are a homogenous group and validation of predictive factors could be useful to stratify patients to receive investigational approaches [5–6].

The purpose of the study was to identify the main predictive factors of pCR after nCRT in LARC that could be used in the next future for treatment decision making.

## MATERIALS AND METHODS

### Patient selection

Medical records of consecutive patients with LARC treated with nCRT between January 2008 and June 2014 were retrospectively reviewed. The study was approved by the Institutional Gastro-Intestinal Tumor Board at the Policlinico Umberto I, “Sapienza” University of Rome. All patients signed an informed consent. Patients were clinically staged on digito-rectal examination, endorectal ultrasound, chest, abdomen and pelvis computed tomography (CT) and/or pelvic magnetic resonance imaging (MRI) as T3-4 and/or with positive regional lymph- node, without any evidence of distant metastases. All patients had biopsy-proven rectal adenocarcinoma. Exclusion criteria included synchronous tumors, cardiovascular disease, history of neurological or psychiatric disorders, or previous pelvic radiotherapy.

### Treatment

All patients were treated with a long course of nCRT. Radiotherapy (RT) was delivered with a 3D-conformational multiple field technique at a dose of 45 Gy (1,8 Gy/fraction) to the whole pelvis plus a 5,4–9 Gy (1,8 Gy/fraction) to the tumor volume. Chemotherapy (CHT) consisted of weekly OXP (50 mg/m^2^, day 1) and five daily continuous infusions of 5-FU (200 mg/m^2^/day) of each week of RT, based on promising results in high rate of pCR [7–8]. Surgery was planned 7–9 weeks after the end of nCRT and its type was left to surgeon's discretion.

### Data collection

Data collected included: demographics (sex, age, PS, BMI, comorbidities), initial diagnosis and tumor extension (T dimension, TNM classification, grading, tumor distance from anal verge), treatment modalities (both total and daily doses of RT, number cycles of concomitant CHT, interruption CHT, time interval between nCRT and surgery).

The presence of diabetes, pulmonary or hepatic disease was considered as comorbidity.

T dimension was evaluated on endoscopic examination. It was expressed in centimeters (cm) and a cut-off of 5 cm was used. The rationale behind using 5 cm as a cut-off for dimension was that this value is used as a cut-off for several dimensional cancer TNM evaluation, and has been demonstrated to be of prognostic value [9–10]. TNM classification was evaluated on CT and/or MRI exams.

pCR was defined as the absence of any residual tumor cells detected in the operative specimen, both at the primary tumor site and regional lymph nodes (ypT0N0Mx).

### Statistical analysis

Standard descriptive statistics were used to evaluate the distribution of each factor. Continuous variables were reported as means (range) and categorical variables as frequencies or percentages. The association between categorical variables was evaluated for significance by chi-square test. Quantitative variables were compared using the Mann–Whitney rank test. Variables with a p-value ≤ 0.25 on univariate analysis were included in the logistic regression analysis. In the multivariate regression model, continuous variables were dichotomized. Statistical analyses were performed using RStudio-0.98.1091 software. All reported p values were two-sided, and p-values lower than 0.05 were considered significant.

## RESULTS

### Patient characteristics

Overall 99 patients were reviewed and included in the study. Patient baseline characteristics were presented in Table 1. Median age was 63.8 years (range 38 - 79) and 66 patients (66.7%) were male. The vast majority of patients had positive lymph nodes at diagnosis (n = 80; 80.8%). All patients received same RT total dose, as well as CHT regimen. In total 23 patients (23.2%) achieved pCR while 76 (76.8%) had no-pCR. There were no significant differences between pCR and no-pCR groups in term of age, sex, comobidities, BMI, as well as tumor characteristic including disease stage, tumor size and distance from anal verge.

**Table 1:** Baseline characteristics of patients population

Characteristics	Patient (%)			p-value
	Total	pCR	no-pCR	
Sex				0.67
Male	66 (66.7)	14 (60.9)	52 (68.4)	
Female	33 (33.3)	9 (39.1)	24 (31.6)	
Age				0.66
≤ 70	76 (76.8)	19 (82.6)	57 (75)	
> 70	23 (23.2)	4 (17.4)	19 (25)	
BMI				0.67
≤ 35 kg/m^2^	97 (98)	23 (100)	74 (97.4)	
> 35 kg/m^2^	2 (2)	0 (0)	2 (2.6)	
Comorbidities				0.98
No	51 (51.5)	13 (56.5)	38 (50)	
Yes	48 (48.5)	10 (43.5)	38 (50)	
T classification				0.86
2	2 (2)	1 (4.3)	1 (1.3)	
3	85 (85.9)	19 (82.6)	66 (86.8)	
4	12 (12.1)	3 (13.1)	9 (11.9)	
N classification				0.21
0	19 (19.2)	5 (21.7)	14 (18.4)	
1	39 (39.4)	6 (26.1)	33 (43.4)	
2	41 (41.4)	12 (52.2)	29 (38.2)	
Overall stage				0.22
II	19 (19.2)	5 (21.7)	14 (18.4)	
IIIA	1 (1)	0 (0)	1 (1.3)	
IIIB	42 (42.4)	7 (30.4)	35 (46.1)	
IIIC	37 (37.4)	11 (47.9)	26 (34.2)	
Tumor dimension				0.22
≤ 5 cm	65 (65.7)	18 (78.3)	47 (61.8)	
> 5 cm	34 (34.3)	5 (21.7)	29 (38.2)	
Distance from anal verge				0.28
≤ 5 cm	56 (59.6)	14 (60.9)	42 (55.3)	
> 5 ≤ 8 cm	22 (22.2)	2 (8.7)	20 (26.3)	
> 8 cm	21 (21.2)	7 (30.4)	14 (18.4)	

pCR: pathologic complete response; BMI: body mass index; T: tumor; N: node

### Univariate analysis

On univariate analysis, number of cycles of concomitant CHT (> 4 vs ≤ 4) and suspension CHT (yes vs no) were found to be near significant (p = 0.21 and 0.1 respectively). The time interval between nCRT and surgery was also investigated. It was analyzed as ≤ 8 weeks vs > 8 weeks and there was no significant difference in pCR detection (p = 0.25).

### Multivariate logistic regression analysis

Logistic regression showed that pre-treatment tumor dimension < 5 cm (p = 0.035) was a significant predictor for pCR, whereas clinical N0-1 stage only indicated a positive trend (p = 0.084). The other variables that were evaluated (tumor distance from anal verge, number cycles of concomitant CHT, suspension CHT and time interval between nCRT and surgery) were not significantly associated with pCR. Details are shown in Table 2.

**Table 2:** Logistic regression analysis:predictors of pathologic complete response (pCR)

Variable	OR	95% CI	p-value
Interruption CHT			0.145
yes (no)	3.41	0.45 - 16.66	
Interval between nCRT and S			0.178
> 8 w (≤ 8 w)	2.13	0.56 - 5.61	
T dimension			0.035
> 5 cm (≤ 5 cm)	0.25	0.1 - 3.44	
Nodal status			0.084
N2 (N0-1)	2.65	0.97 - 12.62	
Cycles CHT			0.755
≤ 4 (> 4)	0.71	0.13 - 15.04	
Distance from anal verge			0.494
> 8 (≤ 8 cm)	1.57	0.5 - 25.83	

OR: odds ratio; CI: confidence interval; CHT: chemotherapy; nCRT: neoadjuvant chemoradiotherapy; S: surgery; w: weeks; T: tumor; N: nodes

## DISCUSSION AND CONCLUSIONS

Our results showed that pCR to nCRT in primary lesion in the setting of LARC was achieved in 23.2% of patients. Multivariate data analysis supported that patients with a tumor diameter ≤ 5 cm were more likely to achieve pCR after nCRT (OR 0.25; p-value 0.035), whereas it only demonstrated a positive trend between N stage and pCR. Furthermore no correlation between pCR rate and surgical time interval was found. A small number of concomitant CHT cycles and interrution of CHT during RT treatment were only near associated with pCR in the univariate analysis.

We may ask why these results are interesting.

Over the past decade there has been a substantial progress in LARC management, thanks to optimization of both surgical and RT techniques. Independently of tumor response to neo-adjuvant treatment, the current paradigm is to treat all LARC with trimodality therapy, including RT, CHT and surgery [1].

Recent data support that pCR following nCRT is associated with excellent long-term survival, with low rates of local recurrence and distant failure [11]. This issue has achieved relevance to guide decision-making, because pCR has begun to be considered as surrogate of more conservative treatment approach, including a “wait and see” policy, in selected cases [1, 8].

The median pCR rate is 16.5% (range 13% - 22.2%) in the main randomized phase III trials of LARC treated with nCRT [12–15]. Our pCR incidence is at the higher end of the range. It should be probably related to the high responsiveness to nCRT scheme used, with the addition of weekly oxaliplatin to the standard fluorouracil nCRT regime. Indirectly it should be also associated with a dose-level relationship between concomitant CHT and pCR, leading to the detention of near significant predictive value of CHT parameters in the univariate analysis. However the robustness of this hypothesis needs to be confirmed, considering that only the German CAO/ARO/AIO-04 trial reported a higher pCR rates when oxaliplatin was added to standard nCRT (17% vs 13%; p = 0.038) [12].

Currently there is no evidence on which clinical parameter confers a pCR advantage after nCRT. Several studies have attempted to identify potential predictive factors associated with pCR [16–20]. Details are shown in Table 3. Smaller tumor size was the most common factor related to an increased rate of pCR.

**Table 3:** Predicors of pCR in LARC patients receiving nCRT

Author	Study charactecistic			Pathologic complete response (pCR)	
	Type	N patient	Treatment	Rate (%)	Predictors (multivariate analysis)
Das [15]	retrospective	562	nCRT + S	19.2	circumferential extent
Kalady [16]	retrospective	242	nCRT + S	11.6	time interval between nCRT and S
Park [17]	retrospective	544	nCRT + S	12.9	pre-nCRT T mobility, post-nCRT size, post-nCRT morphology
Garland [14]	retrospective	297	nCRT + S	11.4	T size, pre-nCRT N stage
Wallin [18]	retrospective	469	nCRT + S	20.5	CEA level, CHT interruption

N: number; nCRT: neoadjuvant chemoradiotherapy; S: surgery; T: tumor; N: lmph nodes; CEA: carcino embryonic antigen; CHT: chemotherapy

Our results were consistent with these data of the recent medical literature. Garland et al [16] evaluated factors of pCR in 297 patients receiving nCRT and clinical tumor size was found to be independent predictor for pCR (p = 0.036). Tumor size was assessed by endoscopist at the time of pre-treatment colonoscopy and was scaled in discrete intervals of < 3.5 cm, 3.5-7 cm and > 7 cm. Statement on tumor size scale selection was not reported. The majority of patients (58.4%) had lesion of 3.5-7 cm.

Pre-nCRT size (p = 0.001) was identified to be univariate predictor for pCR in Park et al cohort of 249 patients [19]. Pre-nCRT tumor size was assessed using digito-rectal examination and any available colonoscopic or radiologic imaging results. The cut-off point of size stratification was set at 4 cm (≤ 4 cm vs > 4 cm), but its selection criterion was not specified. Nevertheless the true effect-size significance could be questionable, due to the different scale used to stratified pretreatment lesion.Although we looked at different cut-off, results remained comparable: greater tumor diameter predicts lower pCR.

Clinical nodal status at diagnosis was incorporated in the multivariate analysis. The OR for pretreatment nodal stage > N1 was 2,65 (95% CI 0.97 – 12.62). Thus patients with N2 category of disease were about 1.5 time less likely to achieve pCR after nCRT than those with metastasis in 1-3 regional lymph nodes. Because of the wide confidence interval for this finding, conclusions about the effect of nodal involvement at diagnosis was difficult to make. Maybe the trend was not statistically significant probably because of small sample size. Otherwise if we compare our results with those of other series that evaluated the prognostic impact of nodal status in pCR, data seem to strongly suggest that nodal status affect pCR rate. In their retrospective analysis, Garland et al. reported an OR for pretreatment clinical nodal status of 4.38 (95% CI 1.01-19.02), which is similar to our findings [16]. Surely the role of imaging modalities to assess lymph nodes involvement is prone to bias. Accurate N stage is essential to select patients and predicting clinical N status remains an unresolved problem. A meta-analysis revealed a slightly but not significantly superiority of endoluminal ultrasound over CT and MRI in diagnosing involved nodes [21]. The poor accuracy for lymph nodes assessment at MRI was recently confirmed by Al-Sukhni et al [22]. They performed a systematic review and meta-analysis of 21 studies. Results showed that MRI had 77% sensitivity and 71% specificity for N involvement. However, considering that this setting of patients received nCRT, it is difficult to evaluate a posteriori whether differences in clinical and pathological nodal stage can be attributed to the nCRT alone. Importantly nodal status appears to be predictor of pCR.

In our cohort time interval between nCRT and surgery was not correlated with pCR both on univariate and logistic regression analysis. This is an important result within literature data. Extending the interval between nCRT and surgery did not significantly increase the proportion of patients achieving pCR [23–25]. By contrast a recent systematic review showed a probably benefit in prolonging the time interval but a statistical analysis was not performed due to many differences between studies included, with a large degree of variation in interval length [26]. One meta-analysis showed that patients operated after 6-8 weeks had an estimated increase in the relative risk of pCR by 42%, but it was a retrospective data meta-analysis and thus results should be taken with caution [27]. Indeed there are no solid data to confirm the real effect of delaying surgery on clinical practice and discrepancy may be explained by arbitrary selection of nCRT-surgery intervals. Further prospective randomized trials are the only way to answer the question of time interval and its correlationon oncologic outcomes.

This study was limited because it was a single-institute study with a small sample size. Serum CEA level was not considered in our analysis, because this parameter is susceptible to inter-laboratory variations and not routinely recorded at our Department. On the other hand homogeneity in patients population data, as well as in treatment approach represent the principal analysis force. Results should be interpreted cautiously due to the retrospective study nature. That tumor size represents a robust predictive pCR factor remains to be shown definitively. Anyway a combination of retrospective literature data may be required to have the most clinical utility in LARC management.

This study demonstrated an association between tumor diameter ≤ 5 cm and pCR in LARC patients treated with nCRT. Time interval between the end of nCRT and surgery was not associated with pCR. Randomized trials are need. Waiting for those data, our results should contribute to develop individualized treatment approach.
